# Host genetics influence the rumen microbiota and heritable rumen microbial features associate with feed efficiency in cattle

**DOI:** 10.1186/s40168-019-0699-1

**Published:** 2019-06-13

**Authors:** Fuyong Li, Changxi Li, Yanhong Chen, Junhong Liu, Chunyan Zhang, Barry Irving, Carolyn Fitzsimmons, Graham Plastow, Le Luo Guan

**Affiliations:** 1 Department of Agricultural, Food and Nutritional Science, University of Alberta, Edmonton, Alberta T6G 2P5 Canada; 2 Lacombe Research and Development Centre, Agriculture and Agri-Food Canada, Lacombe, Alberta T4L 1W1 Canada

**Keywords:** Cattle, Feed efficacy, Heritability, Host genotype, Rumen microbiota

## Abstract

**Background:**

The symbiotic rumen microbiota is essential for the digestion of plant fibers and contributes to the variation of production and health traits in ruminants. However, to date, the heritability of rumen microbial features and host genetic components associated with the rumen microbiota, as well as whether such genetic components are animal performance relevant, are largely unknown.

**Results:**

In the present study, we assessed rumen microbiota from a cohort of 709 beef cattle and showed that multiple factors including breed, sex, and diet drove the variation of rumen microbiota among animals. The diversity indices, the relative abundance of ~ 34% of microbial taxa (59 out of 174), and the copy number of total bacteria had a heritability estimate (*h*^2^) ≥ 0.15, suggesting that they are heritable elements affected by host additive genetics. These moderately heritable rumen microbial features were also found to be associated with host feed efficiency traits and rumen metabolic measures (volatile fatty acids). Moreover, 19 single nucleotide polymorphisms (SNPs) located on 12 bovine chromosomes were found to be associated with 14 (12 of them had *h*^2^ ≥ 0.15) rumen microbial taxa, and five of these SNPs were known quantitative trait loci for feed efficiency in cattle.

**Conclusions:**

These findings suggest that some rumen microbial features are heritable and could be influenced by host genetics, highlighting a potential to manipulate and obtain a desirable and efficient rumen microbiota using genetic selection and breeding. It could be a useful strategy to further improve feed efficiency and optimize rumen fermentation through targeting both cattle and their rumen microbiota.

**Electronic supplementary material:**

The online version of this article (10.1186/s40168-019-0699-1) contains supplementary material, which is available to authorized users.

## Background

Ruminants have evolved to possess a diverse symbiotic microbiota in their rumen, mainly consisting of bacteria, archaea, ciliated protozoa, fungi, and viruses [1]. These rumen microorganisms can degrade complex plant fibers and polysaccharides and produce volatile fatty acids (VFAs), microbial proteins, and vitamins, which provide nutrients to meet the host’s requirement for maintenance and growth. Using omics-based approaches, recent studies have suggested that differences in rumen microbiota are associated with cattle production and health traits, such as feed efficiency [2, 3], methane (CH_4_) yield [4, 5], milk composition [6], and ruminal acidosis [7]. Hence, the rumen microbiota is a potential target for manipulation to improve ruminant productivity and animal health, as well as to reduce CH_4_ emissions.

Although it has been commonly accepted that diet plays the main role in shaping the gut microbiota [8], more and more evidence from quantitative genetics, especially genome-wide association studies (GWAS), have revealed that host genetics is also an important factor in determining the composition of gut microbiota in humans and mice. For example, 18 quantitative trait loci (QTLs) were found to be associated with the abundance of gut microbial taxa in mice [9], and a follow-up study reported 42 QTLs for the abundance of 39 microbial taxa in a different mouse strain [10]. Two studies found that the abundance of one-third of the identified operational taxonomic units (OTUs) in human gut was heritable with moderate or high heritability estimates [11, 12]. In addition, substantial associations between specific host genes and the gut microbiota were observed in the UK human population using GWAS [12]. Recently, 58 single nucleotide polymorphisms (SNPs) were also reported to be associated with the abundance of 33 fecal microbial taxa in human [13].

In ruminants, studies have also indicated that the rumen microbiota could be influenced by host breed/species [14–19]. For instance, differences in the composition of rumen microbiota were detected between Holstein and Jersey dairy cows fed the same diet [18]. However, multiple factors are confounded in that study such as lactation cycles and age, and these factors have been reported to contribute to the variation of the rumen microbiota [20, 21]. In one recent study investigating the role of rumen microbiota in CH_4_ emissions, host genetics was reported to affect the archaea to bacteria ratio in the rumen [17], but it was unclear whether host genetics affect the rumen microbial composition. In another survey of rumen microbiota of 742 rumen and foregut samples from 32 species/sub-species of ruminants and foregut fermenters across continents [15], the identified effects of diet, geographical regions, and genetic background of the host were nested and could not be clearly separated. Effects of breed [14] and sire breed [16] on the rumen microbiota were observed in our previous studies investigating beef cattle, when rumen microbial communities were characterized using low-resolution methods (PCR-DGGE and qPCR). Our recent studies reported individualized rumen microbiome of beef cattle even when animals were fed the same diet and managed under the same environment, and identified breed effect on active rumen microbiome using metagenomics and/or metatranscriptomics [2, 19]. In addition, it is notable that heritability estimates of rumen bacterial and archaeal members were recently reported based on 750 lactating Holstein cows from five commercial herds [5]. All these findings suggest the important role of host genetics in influencing the rumen microbiota. However, the heritability estimates of rumen microbial features for commercial beef cattle and underlying bovine genotypes associated with these microbial features have not been reported. The lack of such information could be one of the barriers to manipulating the rumen microbiota to improve feed efficiency in beef cattle.

Therefore, in this study, we hypothesized that rumen microbial features of beef cattle are affected by host additive genetic effects and there are host SNPs contributing to the variation of microbial composition in the rumen, which could partially drive the “individualized” rumen microbiota and influence host feed efficiency. To test these hypotheses, we assessed compositional profiles of rumen microbiota, estimated the heritability, performed GWAS for rumen microbial features, and correlated these heritable microbial features to feed efficiency traits through surveying a cohort of beef cattle (*n* = 709) raised under the same farm environment.

## Methods

### Animal experiments and rumen sampling

A total of 709 beef cattle from three breeds, including purebred Angus (ANG, *n* = 203) and Charolais (CHAR, *n* = 114) cattle, and the Kinsella composite hybrid (HYB, *n* = 392), were raised under the same feedlot conditions at the Roy Berg Kinsella Research Ranch at the University of Alberta. The Kinsella composite hybrid (HYB) population was bred from multiple beef breeds including Angus, Charolais, Galloway, Hereford, Holstein, Brown Swiss, and Simmental as described previously [22]. The experimental protocol was developed according to the guideline of the Canadian Council on Animal Care [23] and was approved by the Animal Care and Use Committee of the University of Alberta (protocol no. AUP00000882). Animals were fed with different diets according to their breed, sex, and growth stages, and such information was recorded and shown in Additional file 1: (Table S1) and Additional file 2: (Table S2). When the cattle were 292.9 ± 0.6 (mean ± SEM) days of age, approximately 50 ml of rumen sample (including rumen fluid and feed particles) was collected from each animal using oro-gastric tubing before feeding as previously described [24]. Samples were immediately frozen using dry ice and then stored at − 80 °C for further processing. VFA profiling was conducted for each rumen sample using gas chromatography (GC) following the procedures described previously [14], and profiles were successfully obtained for 708 samples (Additional file 1: Table S1). Feed efficiency phenotypes, including dry matter intake (DMI), average daily gain (ADG), residual feed intake (RFI), and feed conversion ratio (FCR), were recorded for a total of 572 cattle (*n* = 184 for ANG, *n* = 91 for CHAR, and *n* = 297 for HYB; Additional file 1: Table S1) among the whole cohort. Briefly, dry matter intake (DMI) values were individually recorded using the GrowSafe system (GrowSafe Systems Ltd., Airdrie, AB, Canada). Residual feed intake (RFI) values were calculated based on DMI, ADG, metabolic weight (MWT) and were further adjusted for backfat thickness (RFIf) as described by Basarab et al. [25]. Feed conversion ratio (FCR) was calculated as the ratio between DMI and ADG. Individual phenotypes and metadata are listed in (Additional file 1: Table S1), and descriptive statistics of feed efficiency phenotypes and VFA concentrations are summarized in (Additional file 3: Table S3).

### DNA extraction, high-throughput sequencing, and quantitative PCR (qPCR) analysis

Total DNA was isolated from each rumen sample using QIAGEN BioSprint 96 workstation (Valencia, CA, United States) at Delta Genomics (Edmonton, AB, Canada). To assess the rumen microbial profiles, the bacterial V1–V3 region and the archaeal V6–V8 region of 16S rRNA genes were amplified using primers as described previously [15], i.e., for bacteria, the primers were Ba9F (5′-GAGTTTGATCMTGGCTCAG-3′) and Ba515Rmod1 (5′-CCGCGGCKGCTGGCAC-3′); for archaea, the primers were Ar915aF (5′-AGGAATTGGCGGGGGAGCAC-3′) and Ar1386R (5′-GCGGTGTGTGCAAGGAGC-3′). The paired-end sequencing (2 × 300 bp) of regional amplicon was performed using the Illumina MiSeq PE300 at Génome Québec Innovation Centre (McGill University, Montréal, QC, Canada). Quantitative PCR was performed to determine the abundance of rumen bacteria and archaea through enumerating their 16S rRNA gene copy numbers, using U2 primers for bacteria (forward: 5′-ACTCCTACGGGAGGCAG-3′; reverse: 5′-GACTACCAGGGTATCTAATCC-3′) [26] and uniMet1 primers for archaea (forward: 5′-CCGGAGATGGAACCTGAGAC-3′; reverse: 5′-CGGTCTTGCCCAGCTCTTATTC-3′) [27]. Standard curves were made using serial dilutions of plasmid DNA containing a full-length 16S rRNA gene of *Butyrivibrio hungatei* (for U2 primers, using an initial concentration of 8.50 × 10^7^ mol/μl) and partial 16S rRNA gene of *Methanobrevibacter* sp. strain AbM4 (for uniMet1 primers, using an initial concentration of 1.58 × 10^7^ mol/μl). Quantitative PCR was conducted using SYBR Green chemistry (Fast SYBR Green Master Mix; Applied Biosystems) in the StepOnePlus Real-Time PCR System (Applied Biosystems), and the 16S rRNA gene copy numbers per milliliter of rumen sample were calculated using the formula from a previous study [27].

### Microbial composition analysis

Sequencing data were processed using MacQIIME version 1.9.1. Briefly, paired-end forward and reverse reads were joined, and then primers and homopolymer runs (maximum length, 8) of joined sequences were trimmed. Only sequences ≥ 400 bp in length, with average quality score ≥ 25 and with ambiguous bases ≤ 6 remained for downstream analysis. De novo chimera checking was performed using UCHIME [28] and operational taxonomic unit (OTU) picking was conducted using USEARCH [29] to cluster similar sequences sharing ≥ 97% similarity. Representative sequences for bacterial and archaeal OTUs were assigned to the Greengenes 16S rRNA gene database (version gg_13_8) [30] and RIM-DB database [31], respectively, using BLAST [32]. Samples with < 500 bacterial sequences or samples with < 100 archaeal sequences were removed from the compositional analysis [15]. To estimate Good’s coverage and α-diversity indices (Chao1, Shannon index, and Simpson index), the number of bacterial and archaeal sequences per sample were normalized to 2000 and 500, respectively, using 100 subsampling iterations. These α-diversity indices were calculated at the genus level for bacterial communities and at the species level for archaeal communities. β-diversity (Principal Coordinates Analysis [PCoA]) was calculated based on normalized sequence numbers (*n* = 2000 for bacteria and *n* = 500 for archaea) using Bray-Curtis dissimilarity matrices. Samples with read number less than these cutoffs were not included in the diversity analysis. Permutational multivariate ANOVA (Adonis PERMANOVA) based on Bray-Curtis dissimilarity matrices was performed with 1000 permutations to test the differences of rumen microbial communities in the R package vegan (https://CRAN.R-project.org/package=vegan). The sequencing data were collapsed and summarized at five taxonomic levels (from genus to phylum) for bacteria, and at six taxonomic levels (from species to phylum) for archaea. Only taxa with a relative abundance > 0.5% in at least one sample and with a prevalence > 20% were considered as detected taxa and included in the downstream analysis.

### Co-occurrence network of rumen microbiota

Correlations among detected bacterial genus-level taxa and archaeal species-level taxa were inferred using the SparCC program [33] implemented in mothur [34], with default settings apart from “permutations = 10000”. To avoid the potential bias on the co-occurrence calculations caused by different sequencing depth among samples, bacterial and archaeal sequences were subsampled to 2000 and 500 for each sample, respectively, and samples with read number less than these cutoffs were removed from the downstream analysis. Bacterial and archaeal taxa that were found in < 20% of animals in the population were also eliminated as previously suggested [35]. The correlation patterns were further filtered to select only correlations with coefficient > 0.3 or < − 0.3 and with *P* value < 0.001, which were then displayed using Cytoscape [36].

### Genotyping

Genomic DNA was extracted from the ear tissue of each animal, and genotyping was performed for all 709 beef cattle using the Illumina BovineSNP50 v2 Genotyping BeadChip containing 54,609 SNPs (San Diego, CA, USA) at Delta Genomics (Edmonton, AB, Canada). A number of 675 individuals were successfully genotyped with genotypes > 80% (Additional file 1: Table S1). Quality control for SNPs was performed according to the following criteria: (1) *P* value of chi-square test of Hardy-Weinberg equilibrium > 10^−6^, (2) minor allele frequency (MAF) < 5%, and (3) genotyping call rate < 90%. Missing genotypes were imputed using the R package synbreed [37]. After that, 42,809 SNPs remained to construct the genomic relationship matrix (*G*) which was used in an animal model to estimate the heritability. In total, 42,374 SNPs with known chromosomal position were used for GWAS (Additional file 4: Table S4).

### Heritability estimations

Only animals with completed rumen microbial profiles, genotype information, breed, sex, diet, and age records were included in this analysis (Additional file 1: Table S1). The relative abundance value of each microbial taxon was log10-transformed [9]. All values of rumen microbial features were plotted and possible outliers (out of mean ± 3SD) were removed, resulting in a total of *n* = 646~668 animals in the analyses for each microbial feature. To capture the additive genetic relationships among individuals, the genomic relationship matrix (*G*) was constructed based on the SNPs after quality control (*n* = 42,809) using the method previously developed [38] in the R package synbreed [37]. The heritability of each rumen microbial feature was estimated using the following animal model in ASReml [39]:

1 $$ {y}_{\mathrm{ijklm}}=\mu +{b}_i+{s}_j+{d}_k+{g}_l+{a}_m+{e}_{\mathrm{ijklm}} $$

Where *y*_ijklm_ is the microbial feature including log10-transformed abundance, alpha-diversity indices, and the top five bacterial/archaeal PCoAs from the Bray-Curtis matrices based PCoA as listed in Table 1; *μ* is the overall mean; *b* is the fixed breed effect with three classes (ANG, CHAR, and HYB); *s* is the fixed effect explaining differences between bull, heifer, and steer; *d* is the fixed effect of four different diets; *g* is the covariate representing the age effect at sampling, *a* is the random additive genetic effect following a distribution of *N*(0, *Gσ*_*a*_^2^), with the genomic relationship matrix *G* and the additive genetic variance *σ*_*a*_^2^; *e* is the random residual effect following *N*(0, I*σ*_*e*_^2^), with identity matrix I and residual variance *σ*_*e*_^2^. The heritability (*h*^2^) was defined as:

**Table 1:** Heritability estimates of rumen microbial abundance, diversity indices^1^, and ratios between dominant microbial groups

Rumen microbial taxonomic features	Heritability (*h*^2^ ± SE)
Bacteria	
16S rRNA gene copy number (log10)	0.16 ± 0.07
Chao1 index	0.09 ± 0.07
Shannon index	0.23 ± 0.09
Simpson index	0.19 ± 0.08
PCoA1 (6.88% variation)	0.12 ± 0.07
PCoA2 (5.13% variation)	0.25 ± 0.09
PCoA3 (3.33% variation)	0.08 ± 0.06
PCoA4 (2.75% variation)	0.00 ± 0.00
PCoA5 (2.40% variation)	0.15 ± 0.09
Archaea	
16S rRNA gene copy number (log10)	0.05 ± 0.06
Chao1 index	0.00 ± 0.05
Shannon index	0.04 ± 0.06
Simpson index	0.05 ± 0.06
PCoA1 (35.19% variation)	0.17 ± 0.09
PCoA2 (22.31% variation)	0.17 ± 0.08
PCoA3 (6.18% variation)	0.05 ± 0.06
PCoA4 (4.58% variation)	0.00 ± 0.00
PCoA5 (2.76% variation)	0.06 ± 0.06
Ratio^2^	
Archaea to bacteria	0.04 ± 0.06
*Firmicutes* to *Bacteroidetes*	0.15 ± 0.07
*Mbb. gottschalkii* to *Mbb. ruminantium*	0.17 ± 0.08

^1^To estimate these α- and β-diversity indices, the number of bacterial and archaeal sequences per sample were normalized to 2000 and 500, respectively. α-diversity indices were calculated at the genus level for bacterial communities and at the species level for archaeal communities. Principal Coordinates Analysis (PCoA) was conducted using Bray-Curtis dissimilarity matrices

^2^Abundance from qPCR and relative abundance were both log10-transformed before we calculated these ratios

2 $$ {h}^2={\sigma_a}^2/\left({\sigma_a}^2+{\sigma_e}^2\right) $$

### Genome-wide association studies (GWAS)

Firstly, microbial taxonomic features were adjusted for the fixed effects and covariate, including *breed*, *sex*, *diet*, and *age*. Single nucleotide polymorphism (SNP) positions were obtained using the SNPchiMp v.3 web-based tool [40], and only SNPs with known positions (*n* = 42,374) were kept for the analysis. These SNPs were located on 30 *Bos taurus* chromosomes (29 autosomes [BTA] and the X chromosome; Additional file 4: Table S4). GWAS were performed using rrBLUP [41] in R package as the model below:

3 $$ {y^{\ast}}_{\mathrm{ij}}=\mu +{a}_i+{m}_j+{e}_{\mathrm{ij}} $$

Where y^*^_ij_ is the adjusted values of microbial taxonomic features; *a* and *e* is the random additive genetic effect and the random residual effect, respectively, with assumptions of distribution, variance and covariance structure as descripted above in model [a]; *m* is a fixed effect modeling the additive SNP effect. Genotypes were coded as − 1/0/1 for genotype aa/Aa/AA. For each trait, *P* values from testing the SNP effects were adjusted into genome-wide false discovery rates (FDRs) using the Benjamini-Hochberg method [42]. Associations with FDR < 0.1 were considered significant, and associations with 0.1 < FDR < 0.2 were regarded as suggestively significant.

### Correlation analyses among heritable microbial features, feed efficiency, and volatile fatty acids

Relationships among heritable microbial features (e.g., relative abundance of bacterial genera, archaeal species, alpha- and beta-diversity indices, and 16S rRNA gene copy numbers with *h*^*2*^ ≥ 0.15), feed efficiency traits, and VFAs were investigated using Spearman’s rank correlation in R 3.3.1 [43]. Correlations with *P* values lower than 0.05 were considered significant.

## Results

### Survey of rumen microbiota using a large commercial cohort of beef cattle

**Fig. 1:**
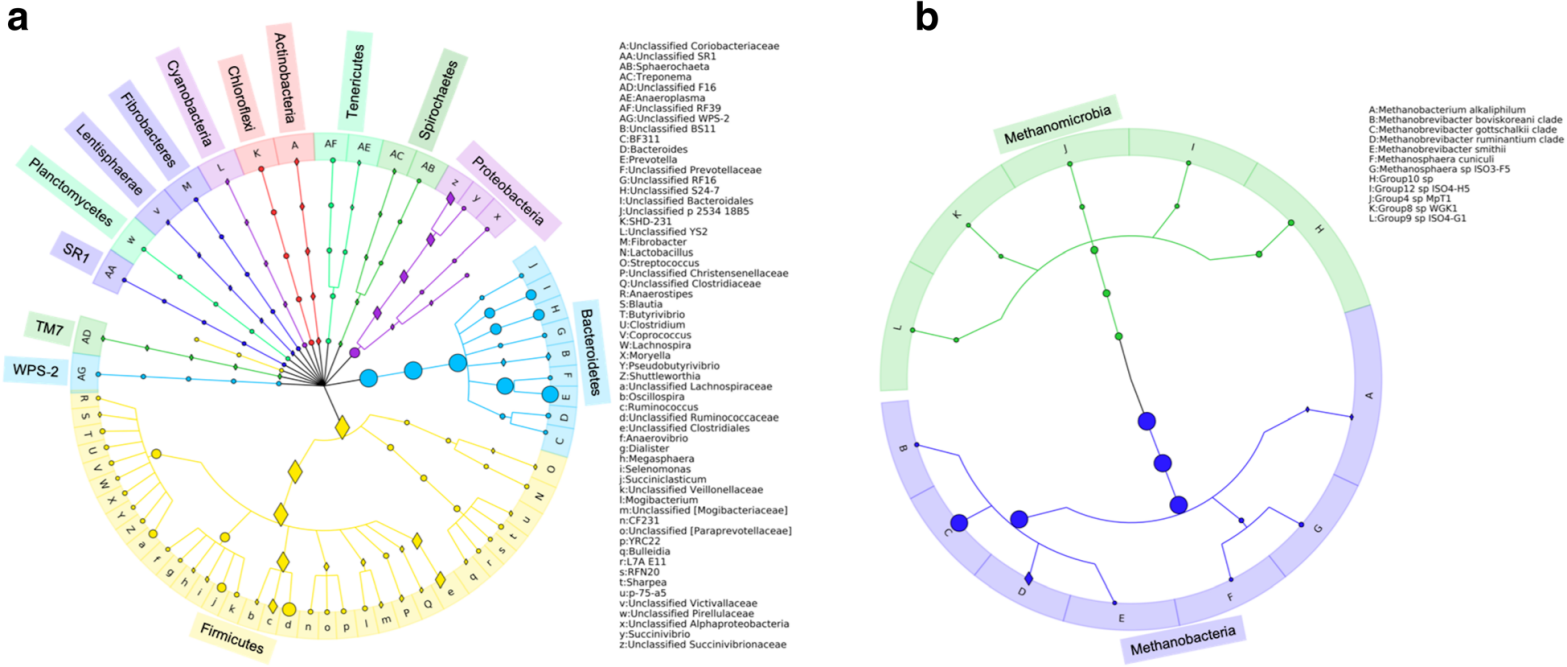
Composition of rumen microbiota in beef cattle. Bacterial community composition was summarized at genus, family, order, class, and phylum levels (**a**), and archaeal community composition was summarized at species, genus, family, order, and class levels (**b**). Heritable taxa (heritability estimate [*h*^*2*^] ≥ 0.15) were indicated using diamonds. These graphs were created using the program GraPhlAn [44]

Rumen microbiota were surveyed using a cohort consisting of bulls (*n* = 71), heifers (*n* = 347), and steers (*n* = 291) that were born in 2013 and raised at the Roy Berg Kinsella Research Ranch at the University of Alberta. Bacterial and archaeal profiles were successfully generated for 668 and 669 animals (with completed records for breed, sex, diet, age, and genotype information), respectively (Additional file 1: Table S1). An average of 8020 ± 98 (mean ± SE) and 1866 ± 22 quality-filtered sequences were generated per animal for bacteria and for archaea, respectively. Good’s coverages for both bacterial and archaeal communities were higher than 99% (Additional file 5: Table S5). After classifying and collapsing these OTUs into different taxonomic levels, 15 phylum-level taxa, 18 class-level taxa, 21 order-level taxa, 34 family-level taxa, and 59 genus-level taxa were detected for bacterial communities (with the relative abundance > 0.5% in at least one sample and with prevalence > 20%), representing 87.10 ± 0.17% of total bacterial reads. Meanwhile, taxa belonged to one phylum, two classes, two orders, two families, eight genera, and 12 species were detected for archaeal communities (Additional file 6: Table S6), representing 99.94 ± 0.01% of total archaeal reads. The dominant bacterial phyla were *Bacteroidetes* (44.05%), *Firmicutes* (36.42%), and *Proteobacteria* (4.61%), and each of the remaining 12 minor phyla accounted for < 1.00% of abundance. The most abundant archaeal taxa were *Methanobrevibacter gottschalkii* (85.09%) and *Methanobrevibacter ruminantium* (9.91%), followed by members of *Methanomassiliicoccaceae* (3.49%) (Fig. 1 and Additional file 6: Table S6). From those 59 bacterial genus-level taxa and 12 archaeal species-level taxa, *Prevotella*, unclassified *Ruminococcaceae*, unclassified *Clostridiales*, unclassified *Bacteroidales*, unclassified *Lachnospiraceae*, unclassified S24-7, and *Methanobrevibacter gottschalkii* were found in all of the animals, representing a core rumen microbiota in beef cattle.

### Breed, sex, and diet drove the segregation of rumen microbiota

**Fig. 2:**
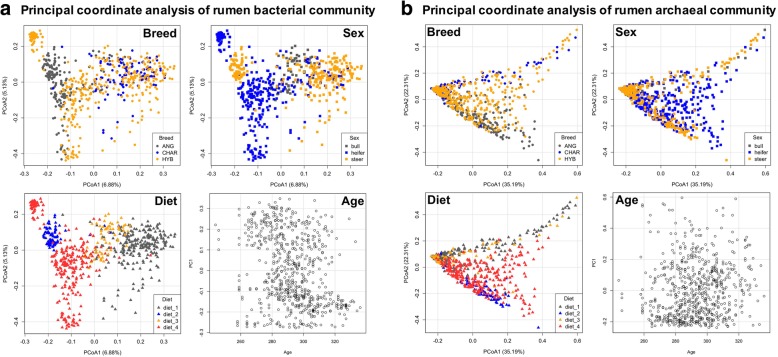
Factors (breed, sex, diet, and age) drive segregation of rumen bacterial communities (**a**) and archaeal communities (**b**), as visualized using principal coordinate analysis (PCoA). To performed PCoA, the number of bacterial and archaeal sequences per sample were normalized to 2000 and 500, respectively, and the PCoA was conducted using Bray-Curtis dissimilarity matrices

**Fig. 3:**
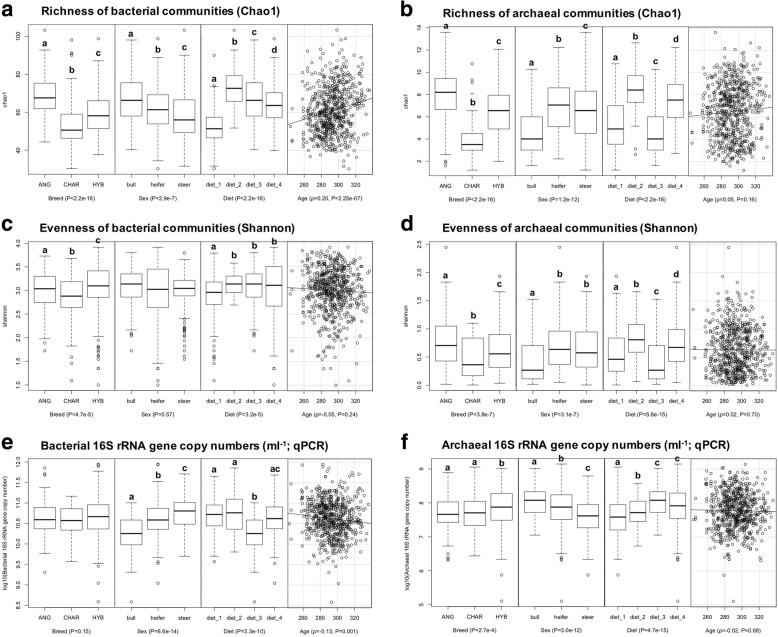
Effects of breed, sex, diet, and age on the richness (**a**, **b**), evenness (**c**, **d**), and abundance (**e**, **f**) of rumen bacteria and archaea. The 16S rRNA gene copy numbers per milliliter of rumen sample were log10-transformed before statistical analysis. Values within each factor that do not have a common superscript are significantly different (*P* < 0.05) according to the Kruskal-Wallis rank sum test. The correlations between age and other indices were calculated using the Spearman’s rank correlation (*ρ* = correlation coefficient)

General community structures (Principal Coordinates Analysis [PCoA] based on Bray-Curtis dissimilarity metrics), alpha-diversity indices (Chao1 for richness and Shannon for evenness), and abundance (16S rRNA gene copy numbers from qPCR) of rumen bacterial and archaeal communities were affected by breed, sex, and diet, while the age effect was only detected for the richness and abundance of bacteria (Figs. 2 and 3 and Additional file 6: Table S6). From 174 detected bacterial and archaeal taxa, 54% (94), 95% (165), 91% (158), and 9% (16) of them were affected by breed, sex, diet, and age (*P* < 0.05 from the animal model), respectively (Additional file 6: Table S6).

Specific to the observed breed effect, both bacterial and archaeal profiles differed between ANG and CHAR breeds of cattle, while those from the HYB were overlapped with the two pure breeds (*P* < 0.05 for both bacterial and archaeal communities based on Adonis permutational multivariate ANOVA [PERMANOVA]; Fig. 2). Charolais rumen microbiota (bacterial and archaeal) were less diverse (with the lowest Chao1 and Shannon indices) than those of ANG and HYB (Fig. 3a–d; *P* < 0.05 according to the Kruskal-Wallis rank sum test), while ANG microbiota had the highest richness (Chao1, *P* < 0.05; Fig. 3a, b). Meanwhile, a similar level of bacterial abundance was detected among the three breed populations (*P* = 0.15 according to ANOVA based on log10-transformed 16S rRNA gene copy numbers per milliliter of rumen sample), with higher archaeal abundance for HYB compared with those in CHAR and ANG (*P* = 2.7e−4; Fig. 3e, f).

Principal Coordinates Analysis also displayed the sex effect on both bacterial and archaeal communities (*P* < 0.05 according to PERMANOVA; Fig. 2). In addition, comparison analysis of alpha diversities revealed that the bull rumen microbiota had the lowest richness and evenness for archaeal communities and highest richness for bacterial communities (*P* < 0.05 according to the Kruskal-Wallis rank sum test; Fig. 3a–d). Among the three sexes, bulls had the highest archaeal but lowest bacterial abundance, while steers had the lowest archaeal but highest bacterial abundance (Fig. 3e, f; *P* < 0.05 according to ANOVA).

### Host additive genetic effects had measurable impact on the rumen microbiota

The proportion of rumen microbial taxon at multiple taxonomic levels was treated as an individual trait as suggested previously [13], and its heritability (*h*^*2*^) was estimated using an animal model based on the genomic relationship matrix (*G* matrix). In the present study, only microbial taxonomic features with the heritability estimate of *h*^*2*^ ≥ 0.15 were considered as being heritable. The results showed that animal additive genetic variation contributed to relative abundance of 59 (56 for bacteria and 3 for archaea) microbial taxa (*h*^*2*^ ≥ 0.15; Fig. 1 and Additional file 6: Table S6) belonging to various taxonomic levels. Among those 59 heritable bacterial taxa, 22 of them belonged to the phylum *Firmicutes*, including *Ruminococcus* (*h*^*2*^ = 0.16 ± 0.08; mean ± SE), unclassified *Clostridiales* (*h*^*2*^ = 0.25 ± 0.09), *Blautia* (*h*^*2*^ = 0.18 ± 0.08), etc. However, most members belonging to *Bacteroidetes*, such as *Prevotella*, unclassified S24-7, and unclassified *Bacteroidales*, were less affected by host genetics (*h*^*2*^ < 0.15). For the three heritable archaeal taxa, the heritability estimate was 0.23 ± 0.08 for *Methanobacterium*, 0.18 ± 0.08 for *Mbb. ruminantium*, and 0.23 ± 0.08 for *Methanobacterium alkaliphilum*.

**Fig. 4:**
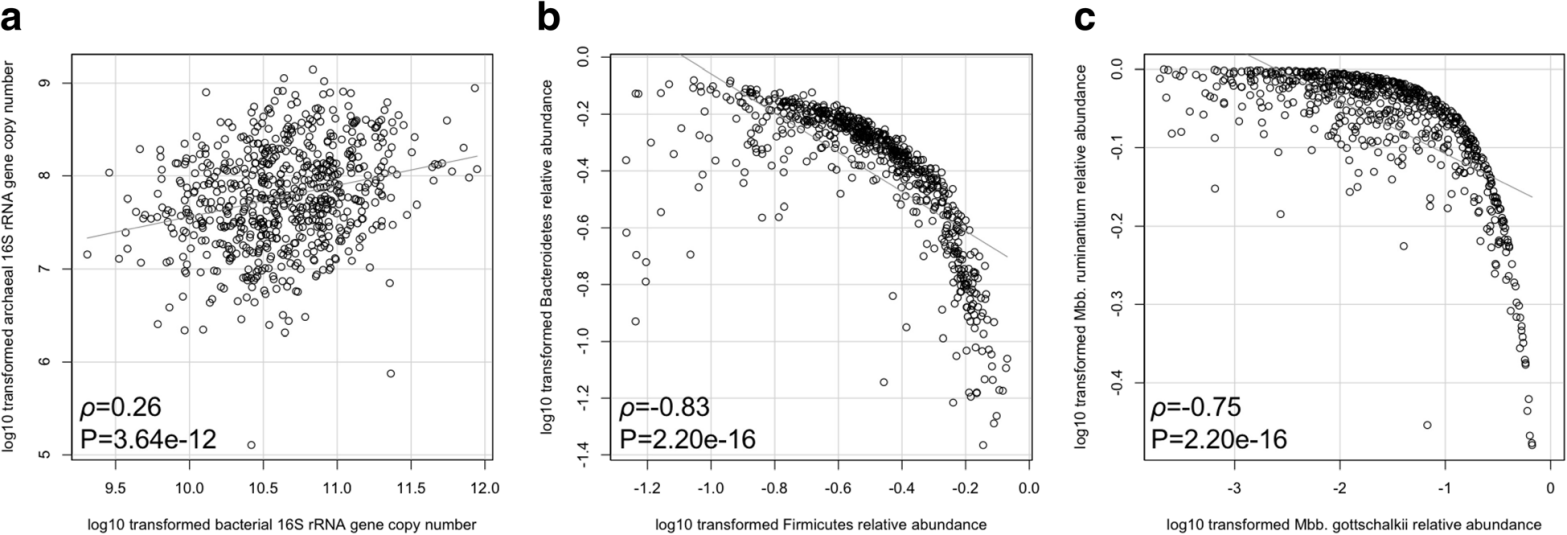
Relationships between predominant rumen microbial groups. **a** Ratio of bacterial abundance to archaeal abundance, represented by 16S rRNA gene copy number obtained using qPCR. **b** Ratio of *Firmicutes* to *Bacteroidetes*. **c** Ratio of *Methanobrevibacter gottschalkii* to *Methanobrevibacter ruminantium*. The 16S rRNA gene copy number and relative abundance were log10-transformed, and the correlation analysis was performed using the Spearman’s rank correlation (*ρ* = correlation coefficient)

In addition, rumen bacterial diversity indices, including Shannon index (*h*^*2*^ = 0.23 ± 0.09) and Simpson index (*h*^*2*^ = 0.19 ± 0.08), were also heritable (Table 1). Meanwhile, moderate heritability estimates (*h*^*2*^ = 0.15~0.25) were obtained for PCoA2 (5.13% of variation) and PCoA5 (2.40% of variation) of bacterial communities and for PCoA1 and PCoA2 (35.19% and 22.31% of variation, respectively) of archaeal communities (Table 1). Moderate heritability was observed for the bacterial abundance (*h*^*2*^ = 0.16 ± 0.07) but not for the archaeal abundance (*h*^*2*^ = 0.05 ± 0.06) (Table 1). Due to correlations between bacterial and archaeal abundances (correlation coefficient [*ρ*] = 0.26, *P* = 3.64e−12; Spearman’s rank correlation), between *Firmicutes* and *Bacteroidetes* (*ρ* = − 0.83, *P* = 2.20e−16;) and between *Mbb. gottschalkii* and *Mbb. ruminantium* (*ρ* = − 0.75, *P* = 2.20e−16) (Fig. 4), host genetics effects on these ratios were also estimated. The ratio between *Firmicutes* and *Bacteroidetes* (*h*^*2*^ = 0.15 ± 0.07), and the ratio between *Mbb. gottschalkii* and *Mbb. ruminantium* (*h*^*2*^ = 0.17 ± 0.08) were both moderately heritable (Table 1).

### Heritable microbial taxa were keystone members of the rumen microbial co-occurrence network

**Fig. 5:**
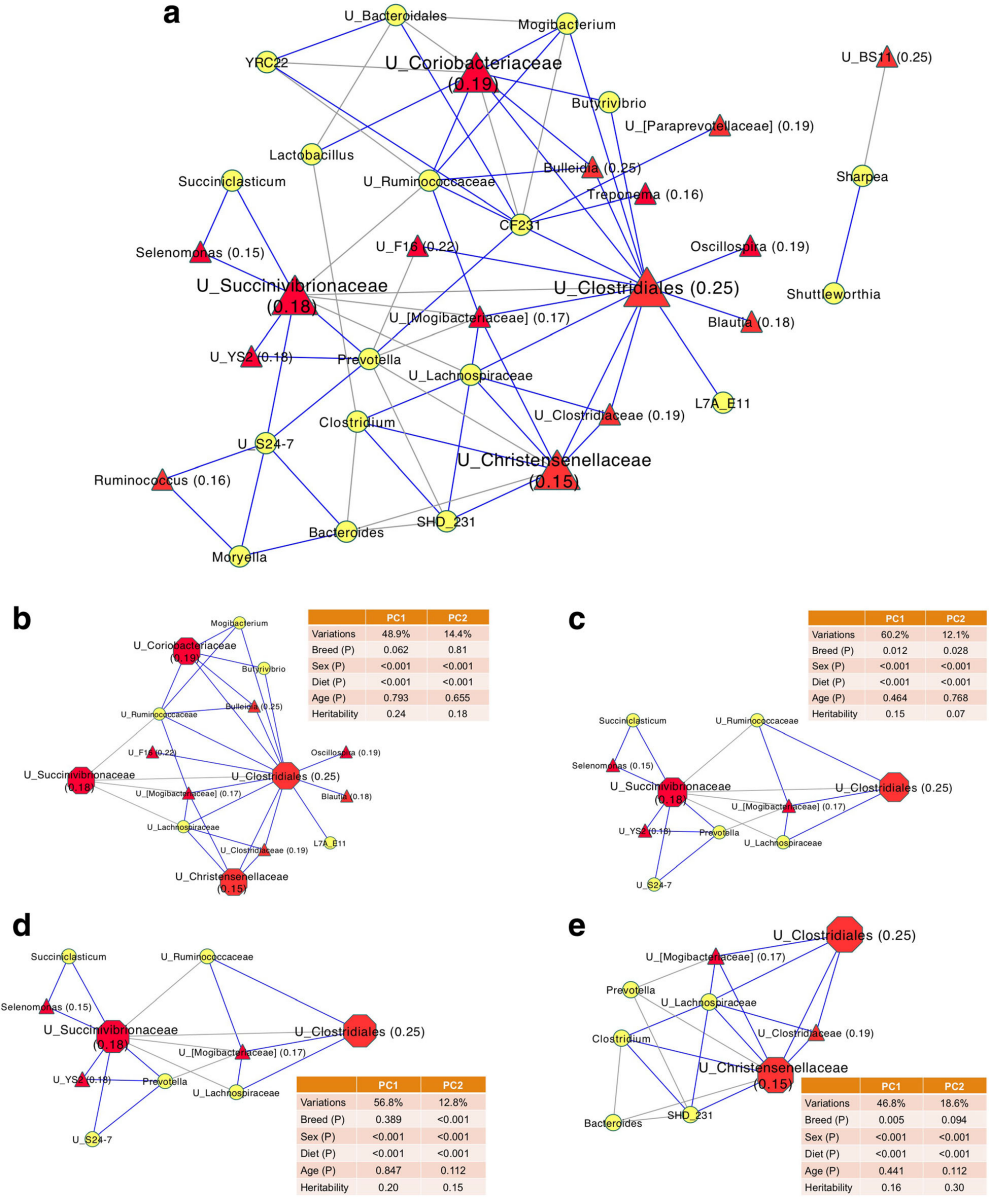
Co-occurrence network of rumen microbial taxa (**a**). Four major co-occurrence network modules were centered by unclassified *Clostridiales* (**b**), unclassified *Succinivibrionaceae* (**c**), unclassified *Coriobacteriaceae* (**d**), and unclassified *Christensenellaceae* (**e**). Only correlations with coefficient > 0.3 or < −0.3 and with *P* value < 0.001 were displayed. Heritable taxa were represented by red triangle/hexagon, while inheritable taxa were represented by yellow circle. Values in the parentheses are heritability estimates of heritable taxa. A connection with a blue/gray line means a positive/negative correlation. ‘*U_*’ before the taxonomic name represents unclassified. The first two PCs were calculated using PCA for each module

Co-occurrence networks were observed for the bacterial communities but not for the archaeal communities (Fig. 5), with 72 significant associations (52 positive and 20 negative) (correlation coefficient < − 0.3 or > 0.3 and *P* < 0.001) being identified between bacterial taxa at the genus level. Four major modules comprised of correlated bacterial taxa were observed, centered by four heritable bacterial taxa (unclassified *Clostridiales*, unclassified *Succinivibrionaceae*, unclassified *Coriobacteriaceae*, and unclassified *Christensenellaceae*, respectively) (Fig. 5b–e).

### Heritable microbial features correlated with host feed efficiency traits and VFAs

**Fig. 6:**
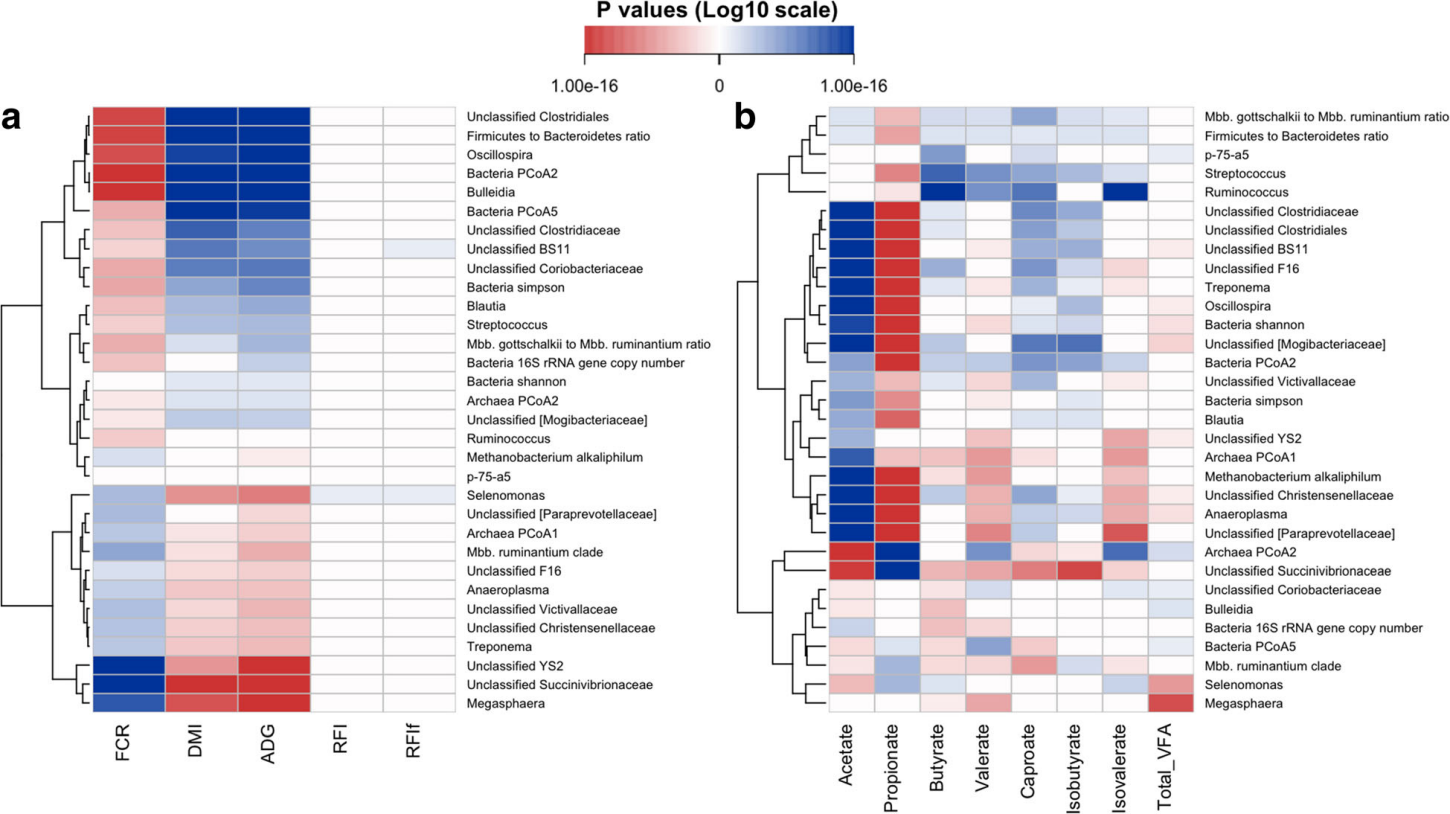
Correlation patterns showing that heritable rumen microbial features (*h*^*2*^ ≥ 0.15) associated with feed efficiency (**a**) and rumen volatile fatty acids (**b**). Correlation analyses were performed using Spearman’s rank correlation, and correlations with *P* values lower than 0.05 were considered significant. Relative abundance of heritable bacterial genera and archaeal species, proportion of VFAs, 16S rRNA gene copy numbers, and ratios were log10-transformed, and possible outliers (out of mean ± 3SD) were removed before the analysis. Negative and positive correlations were displayed in red and blue, respectively

Correlation analysis revealed significant relationships (*P* < 0.05, Spearman’s rank correlation) between rumen microbial features and host feed efficiency traits. Most of heritable microbial features strongly contributed to the variation of FCR, ADG, and DMI but did not relate to RFI or backfat-adjusted RFI (RFIf) (Fig. 6a). Two clusters of heritable microbial features showed strongest correlations with FCR/ADG/DMI (*P* < 1.42e−8). The first cluster included *Bulleidia*, *Oscillospira*, unclassified *Clostridiales*, the *Firmicutes* to *Bacteroidetes* ratio, and bacterial PCoA2, while the second one comprised *Megasphaera*, unclassified *Succinivibrionaceae*, and unclassified YS2. Meanwhile, heritable microbial features were also correlated with major rumen metabolic measures (VFAs), especially with acetate and propionate concentrations (Fig. 6b). For example, unclassified *Clostridiales*, unclassified *Christensenellaceae*, and unclassified *[Mogibacteriaceae]* were positively correlated with acetate and negatively correlated with propionate concentrations, while unclassified *Succinivibrionaceae* was negatively and positively correlated with acetate and propionate concentrations, respectively (*P* < 1.78e−15).

### GWAS identified host SNPs for rumen microbial taxonomic features

**Fig. 7:**
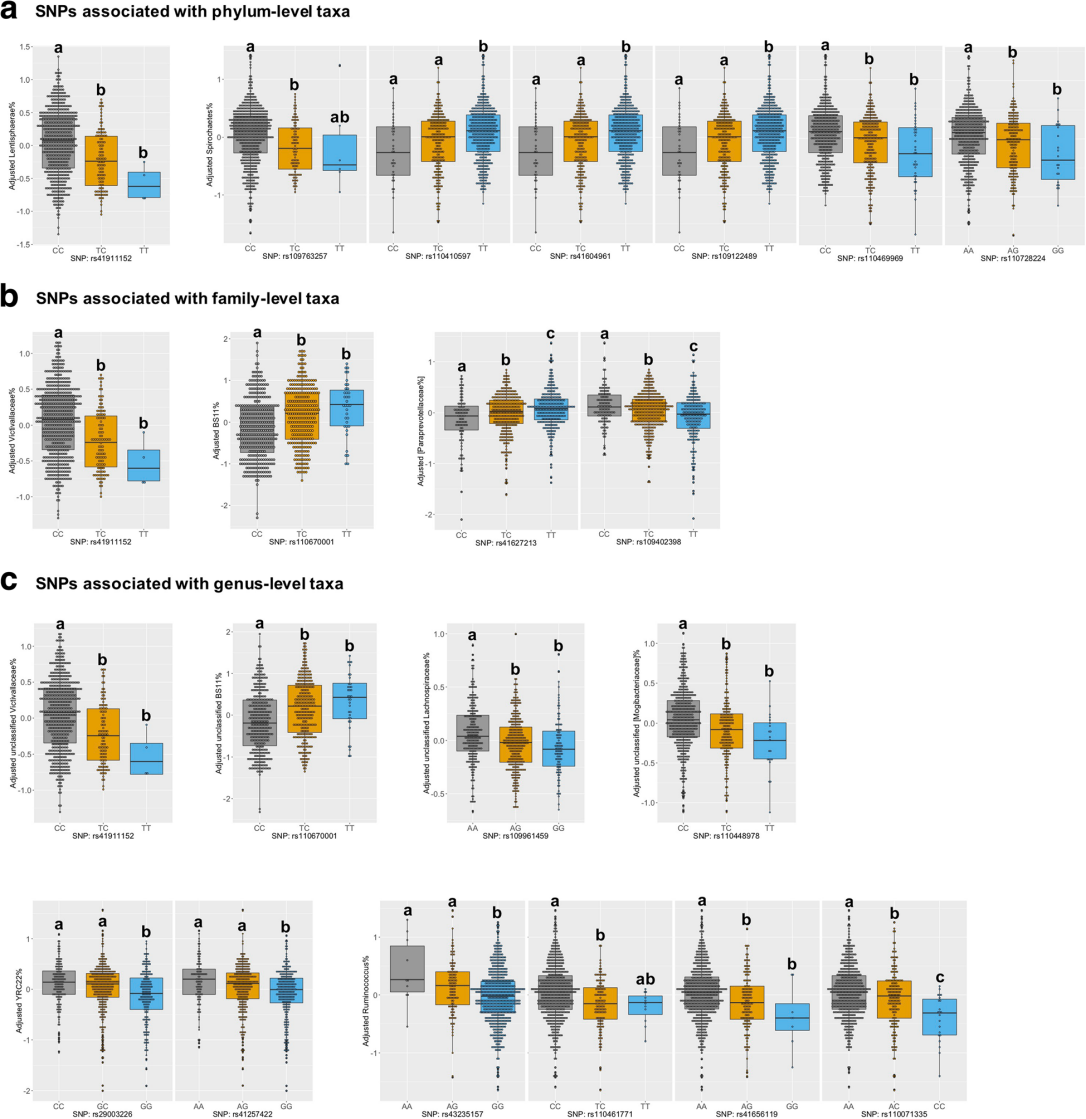
SNPs associated with rumen microbial taxa at phylum (**a**), family (**b**), and genus (**c**) levels. Only associations with false discovery rates (FDR) < 0.1 (significant) and 0.1 < FDR < 0.2 (suggestively significant) are displayed. In each plot, values that do not have a common superscript are significantly different (*P* < 0.05) based on ANOVA. The *x*-axis represents genotype of a SNP, and the *y*-axis indicated the log10-transformed relative abundance after adjusting breed, sex, diet, and age factors

When downstream GWAS were performed for microbial taxonomic features with *h*^*2*^ ≥ 0.1, 19 SNPs located on BTA (*Bos taurus* autosome) 1, 2, 3, 5, 7, 10, 12, 13, 16, 19, 26, and 27 were identified to be associated with microbial taxonomic features at the significance level of false discovery rate (FDR < 0.1) or at the suggestive significance level of 0.1 < FDR < 0.2. Specifically, these SNPs were associated with the abundance of six bacterial genus-level taxa (unclassified BS11, *Ruminococcus*, unclassified *Lachnospiraceae*, YRC22, unclassified *[Mogibacteriaceae]*, and unclassified *Victivallaceae*), three bacterial families (BS11, *[Paraprevotellaceae]*, and *Victivallaceae*), one bacterial order (*Victivallales*), two bacterial classes (*Spirochaetes* and *Lentisphaeria*), and two bacterial phyla (*Spirochaetes* and *Lentisphaerae*) (Table 2 and Fig. 7). No significant (or suggestively significant) association was observed for alpha-diversity indices, PCoAs, bacterial and archaeal abundance, and relative abundance of archaeal taxa.

**Table 2:** Identified bovine SNPs associated with rumen microbial taxa

SNP	Position	Alleles	Gene	Consequence	Associated Taxon	FDR^3^	*P*	FE^4^
rs109763257	1:155345571	C/T	NC region^1^	NA^2^	*Spirochaetes* (phylum)	0.173	1.20e−05	
					*Spirochaetes* (class)	0.190	9.43e−06	
rs43235157	1:156294225	A/G	*TBC1D5*	Intron variant	*Ruminococcus* (genus)	0.191	1.33e−05	DMI
rs110461771	2:92080445	C/T	*RAPH1*	Intron variant	*Ruminococcus* (genus)	0.164	3.88e−06	FCR
rs29003226	3:51976646	C/G	NC region^1^	NA^2^	YRC22 (genus)	0.107	2.53e−06	
rs41257422	5:6266261	A/G	NC region^1^	NA^2^	YRC22 (genus)	0.155	7.33e−06	RFIf
rs41656119	7:83551608	A/G	NC region^1^	NA^2^	*Ruminococcus* (genus)	0.191	1.80e−05	
rs110670001	10:10930797	C/T	NC region^1^	NA^2^	BS11 (family)	0.006	1.43e−07	
					Unclassified BS11 (genus)	0.006	1.43e−07	
rs110071335	10:81981544	A/C	*SMOC1*	Intron variant	*Ruminococcus* (genus)	0.191	1.46e−05	
rs109402398	12:37678844	C/T	NC region^1^	NA^2^	*[Paraprevotellaceae]* (family)	0.105	4.95e−06	
rs110410597	13:28095457	C/T	*OPTN*	Intron variant	*Spirochaetes* (phylum)	0.173	2.45e−05	
					*Spirochaetes* (class)	0.190	2.69e−05	
rs41604961	13:28115879	C/T	*OPTN*	Intron variant	*Spirochaetes* (phylum)	0.173	2.45e−05	
					*Spirochaetes* (class)	0.190	2.69e−05	
rs109122489	13:28149879	C/T	*MCM10*	Intron variant	*Spirochaetes* (phylum)	0.173	2.45e−05	
					*Spirochaetes* (class)	0.190	2.69e−05	
rs110469969	13:28183389	C/T	*UCMA*	Intron variant	*Spirochaetes* (phylum)	0.173	2.45e−05	
					*Spirochaetes* (class)	0.190	2.69e−05	
rs109961459	13:24202640	A/G	NC region^1^	NA^2^	Unclassified *Lachnospiraceae* (genus)	0.111	2.61e−06	
rs41627213	16:78415671	C/T	*DENND1B*	Intron variant	*[Paraprevotellaceae]* (family)	0.070	1.65e−06	
rs41911152	19:30220186	C/T	NC region^1^	NA^2^	*Lentisphaerae* (phylum)	0.070	1.64e−06	DMI
					*Lentisphaeria* (class)	0.070	1.64e−06	
					*Victivallales* (order)	0.034	8.05e−07	
					*Victivallaceae* (family)	0.038	8.92e−07	
					Unclassified *Victivallaceae* (genus)	0.038	8.92e−07	
rs110728224	26:32497450	A/G	NC region^1^	NA^2^	*Spirochaetes* (phylum)	0.173	4.73e−06	
					*Spirochaetes* (class)	0.140	3.31e−06	
rs110448978	26:37871121	C/T	*KCNK18*	Downstream variant	Unclassified *[Mogibacteriaceae]* (genus)	0.187	4.40e−06	ADG DMI FCR
rs42620822	27:42776720	A/G	NC region^1^	NA^2^	*Spirochaetes* (class)	0.196	3.24e−05	

^1^NC region = non-coding region

^2^NA = not available

^3^For each microbial taxonomic feature, *P* value was adjusted into genome-wide false discovery rates (FDRs) using the Benjamini-Hochberg method. Associations with FDR < 0.1 were considered significant, and associations with 0.1 < FDR < 0.2 were regarded as suggestively significant

^4^FE = feed efficiency traits

The most significant associations were BS11 family and unclassified BS11 at the genus level with the SNP: rs110670001 on BTA10 (*P* = 1.43e−07, FDR = 0.006). In addition, four adjacent SNPs (rs110410597, rs41604961, rs109122489, and rs110469969) located in the region of 28.10~ 28.18 Mbp on BTA13, which were in complete linkage disequilibrium (data not shown), tended to be associated with the phylum *Spirochaetes* and the class *Spirochaetes* (*P* = 2.45e−05~2.69e−05, FDR = 0.17~0.19). Moreover, two genus-level taxa (unclassified *Lachnospiraceae* and *Ruminococcus*) tended to be associated with one SNP (rs109961459 on BTA13; *P* = 2.61e−06, FDR = 0.11) and four SNPs (rs43235157 on BTA1, rs110461771 on BTA2, rs41656119 on BTA7, and rs110071335 on BTA10; *P* = 3.88e−06~1.80e−05, FDR = 0.16~0.19), respectively (Table 2 and Fig. 7).

Among those identified SNPs, five of them were also related to feed efficiency traits. Specifically, SNP: rs43235157 (associated with *Ruminococcus*) affected DMI (*P* = 1.64e−03, ANOVA), SNP: rs110461771 (associated with *Ruminococcus*) influenced FCR (*P* = 0.10), SNP: rs41257422 (associated with YRC22) impacted on RFIf (*P* = 5.51e−03), SNP: rs41911152 (associated with unclassified *Victivallaceae*) had an effect on DMI (*P* = 0.08), and SNP: rs110448978 (associated with unclassified *[Mogibacteriaceae]*) related to ADG (*P* = 2.06e−02), DMI (*P* = 4.23e−02), and FCR (*P* = 0.08) (Table 2 and Additional file 7: Figure S1).

## Discussion

Findings from the current study provide answers to some fundamental questions in terms of the rumen microbiota. Firstly, although sex has been suggested as one of the factors affecting the composition of gut microbiota in humans and mice [45, 46], our current study is the first to evaluate the sex effect on the rumen microbiota. This is notable as our study was conducted in a commercial beef cattle operation, and thus cattle with different sexes were fed with different diets to fulfill their different energy requirements. Therefore, the sex effect detected can be nested or confounded with the dietary effect. To take this nested design into consideration, Adonis PERMANOVA was conducted and sex effect was determined through constraining permutations within each diet. This PERMANOVA showed that the sex effect on rumen bacterial and archaeal communities was significant, confirming the sex effect on rumen microbiota. Specifically, we found that the microbiota observed in bulls was distinguishable from that of heifers and steers. A recent study reported that male castration eliminated the gut microbial differences between males and females, and the hormone (e.g., testosterone) treatment prevented the changes of males after gonadectomy [47]. This suggests that differences in sex hormones could be one of the elements to explain the variation among different sexes, because sex hormones affected bile acid profiles [47] and the shifts of bile acid consequently influence the gut microbiota [48]. Meanwhile, males and females may be exposed to different environmental microorganisms due to different diets and different activities [45], and thus it could also in part drive the different microbial profiles between sexes.

Such a sex effect on the rumen microbiota raises several questions, especially in beef cattle. Most of the genetic improvement for productivity was achieved through breeding sires and passing the desirable characteristics to their offspring steers. Our previous study has suggested the sire breed had an effect on the frequency of particular rumen microbial phylotypes in their offspring steers, but the sex factor was not considered [16]. In the current study, three sexes were included for each breed, and sex has now been shown to affect both rumen microbial community structures and relative abundance of many taxa. However, future research on comparing microbiota from multiple generations of beef cattle with different sexes is needed to determine to what extent rumen microbiota in bulls could be passed to their offspring and if this differs for female or male offspring. Recent human studies also highlight the potential vertical transmission of gut microbiota, especially from mothers to infants [49]. Therefore, the magnitude of the dam’s effect on the rumen microbiota also needs to be explored since heifers have different rumen microbiota than bulls.

Secondly, the reported heritability estimates in this study answer the questions to what extent the host genetics can affect the rumen microbiota and whether the host can influence all members at the same level, which provide the theoretical foundation to explain the highly individualized rumen microbiota in cattle. Interestingly, as the predominant bacterial phylum, most of the bacterial taxa (20 out of 22) belonging to *Bacteroidetes* had low heritability estimates, which is consistent with the recent findings based on dairy cows [5]. The low heritability estimates of *Bacteroidetes* members suggest that they are largely affected by environmental factors, such as diet. It has been reported that genes encoding a broad spectrum of carbohydrate-active enzymes (CAZys), especially for glycoside hydrolases (GHs) and glycosyl transferases (GTs), were enriched in *Bacteroidetes* genomes [50]. Moreover, polysaccharide utilization loci (PULs), genomic regions encoding all necessary enzymes for the binding and degradation of plant structural polysaccharides, were identified in 64 culturable *Bacteroidetes* genomes [51], and their high occurrences in *Bacteroidetes* were further confirmed through metagenomic analysis [50], representing a polysaccharide-degradation strategy evolved by *Bacteroidetes*. All these results highlight the essential roles of *Bacteroidetes* members in the degradation and fermentation of plant-structural polysaccharides in the rumen that are the main component of feed materials. Therefore, they are likely to be able to adapt to various diets, and many studies have indeed suggested diet as the major factor determining the abundance of *Bacteroidetes*, *Prevotella*, unclassified *B*acteroidales, and so on [15]. Such results are in line with studies on human gut microbiota, in which taxa belonging *Bacteroidetes* were not heritable and showed obvious shifts under dietary interventions [11, 52]. However, a recent study reported that 15 out of 22 heritable rumen OTUs belonged to *Bacteroidetes* [53], which is inconsistent with our findings. It is notable that they conducted the heritability estimation with only 47 cows [53], and such a small sample size may lead to biased estimations of the host additive genetic effects on the rumen microbiota.

On the other hand, phylum *Firmicutes* (the second most abundant phylum) and many taxa belonging to this phylum (21 out of 52) had moderate heritability estimates, suggesting that the host genetic effect contributes to the observed variation in this phylum. This is also consistent with a previous study of human gut microbiota [12]. For example, as the most abundant family in *Firmicutes*, *Ruminococcaceae* had moderate heritability. This family is composed of both fibrolytic organisms and members involved in starch hydrolysis, which could produce acetate, formate, succinate, and so on [54, 55]. Unclassified *Clostridiales* in this family has been reported to be affected by both host and diet [15], and the moderate heritability estimate obtained in this study further confirmed the host genetic effect on its abundance. Although a previous study indicated that unclassified *Clostridiales* may play a role in biohydrogenation [56], the ecology and functions of phylotypes belonging to this group are largely unknown because most of them are unculturable. Regardless, the observed different heritability estimates between members of *Bacteroidetes* and *Firmicutes* suggest that host effects are not equal on different rumen microbial phylotypes. Therefore, genetic selection and breeding may be applied to alter rumen microbial taxa with moderate heritability estimates, while it is unlikely to have any effects on those members driven by environmental factors.

Coevolution of microorganisms with host might be one of the mechanisms to explain different host genetic effects on different rumen microbial taxa. As described above, we found that the abundance of *Ruminococcus* was influenced by host genetics. It has been reported that members of this genus display large diversity and particular host-association patterns in different mammalian species [57], supporting the suggestion that there are coevolutionary relationships between *Ruminococcus* and the host. In addition, as major butyrate producers (e.g., *Butyrivibrio*, *Clostridium*, etc.) [54], most members of *Lachnospiraceae* (9 out of 10) were not heritable in the rumen, whereas most members of this family were reported to be heritable in the human gut [11]. This inconsistency of heritability estimates of *Lachnospiraceae* members between ruminant and human further suggests there are coevolutionary relationships between host and the gut microbiota. Further scanning and analysis of genomic characters of these heritable rumen taxa, such as the outcomes of the Hungate 1000 project [51] and the 913 microbial genomes assembled from rumen metagenomes [50], will provide more information to explain how host and rumen microorganisms coevolved at the genomic level and provide a better understanding of how host genetics influence these microbial taxa.

Four heritable bacterial taxa (unclassified *Succinivibrionaceae*, unclassified *Clostridiales*, unclassified *Coriobacteriaceae*, and unclassified *Christensenellaceae*) interacted with many other bacterial taxa, suggesting that they may be the keystone members of the rumen microbiota. For instance, members of *Succinivibrionaceae* could utilize hydrogen to generate succinate (a precursor of propionate) [58], thus reducing the H_2_ release and methane emissions. Therefore, they may function as one of the focal points to connect with propionate production, hydrogen utilization, and methanogenesis in the rumen. Indeed, the abundance of members in *Succinivibrionaceae* not only associated with methane emissions [4], but also showed significant correlations with feed efficiency and rumen propionate in the present study. Moreover, it has been reported that there are strong interactions between *Succinivibrionaceae* and other major rumen microorganisms at the transcriptional level [59]. All these above mentioned findings support the suggestion that members of *Succinivibrionaceae* play an essential role in the rumen due to their ecological and metabolic functions. Therefore, the host genotype may directly control these heritable keystone members and indirectly impact the other taxa through the microbial interactions. Future research on isolating and characterizing members of these heritable keystone members could help define their ecological niches in the rumen and reveal mechanisms between their interactions with the host and other rumen microorganisms.

Furthermore, the identification of associations between host genotypes (SNPs) and rumen microorganisms through GWAS provides further answers on which genetic components contribute to the variation of rumen microbiota of beef cattle. For instance, the SNP: rs110461771 (associated with the variation in the abundance of *Ruminococcus*) is located within the gene *RAPH*1 (Ras Association (RalGDS/AF-6) and Pleckstrin Homology Domains 1) on BTA2. The *RAPH*1 gene is involved in cell migration, which is the function that has been suggested to be associated with the nutrient absorption abilities of the rumen epithelia in beef steers [60]. Therefore, polymorphism of the *RAPH*1 gene may contribute to differences in the rumen epithelial absorption of nutrients such as VFAs. The variation in ruminal epithelial VFA absorption has been reported to be associated with differences in ruminal pH [61], and the shift in ruminal pH can influence the rumen microbiota [62]. Another SNP: rs29003226 (associated with the abundance of YRC22) is close to the *CDC*7 (cell division cycle 7) gene on BTA3. The *CDC*7 gene encodes the cell division cycle protein with kinase activity and might be involved in the cell division of the rumen epithelium. It has been reported that increased cell division could increase the proportion of epithelial cells, papillae length, and papillae number [63], and the variation of these rumen physical structures are expected to have a potential influence on the rumen microbiota [17]. In addition, the SNP: rs41911152 (associated with various microbial groups) is located upstream of *MYH*3 (Myosin Heavy Chain 3) on BTA19. The *MYH3* gene plays a role in muscle contraction [64], and thus it may relate to rumen contraction frequency by affecting the muscle action of the rumen wall. Rumen contraction frequency is associated with the passage rate of rumen digesta which has been suggested to also influence the microbiota [17]. Furthermore, expression of all three genes in the rumen epithelial wall were detected in HYB beef steers raised under the same environment in our previous study [60]. Overall, the above microbiota-associated SNPs suggest that the host genetics driven rumen physical features, and gene expression could drive the composition of rumen microbiota. Future follow-up studies to evaluate the associations between these genes and regions (using higher density SNP markers and/or gene sequencing) and rumen epithelial structure and thickness, passage rate, ruminal pH, and rumen microbiota will provide more direct evidence to support our suggestions.

Five rumen microbiota-associated SNPs also contributed to the variation of feed efficiency traits in the current beef cohort, and four of them have already been located in the QTLs for feed efficiency in previous studies (e.g., rs43235157 and rs41257422 in the QTLs for ADG, rs41911152 and rs110448978 in the QTLs for RFI) [65–67]. Some other microbiota-associated SNPs overlap with known quantitative trait loci (QTLs) for feed efficiency as well. For example, SNPs on BTA1 (rs109763257) and BTA13 (rs110410597, rs41604961, rs109122489, and rs110469969) are located within the QTLs for ADG [65, 68]. Meanwhile, SNPs on BTA3 (rs29003226) and BTA26 (rs110728224) overlap with QTLs for RFI [67]. Such overlap suggests that these QTLs may have pleiotropic effects on both rumen microbiota and feed efficiency, which may partly explain the associations between rumen microorganisms and feed efficiency [2, 3]. For instance, a pervious study reported associations between the unclassified [*Mogibacteriaceae*] and feed efficiency [69], and the QTL for feed efficiency on BTA26 overlaps with the SNP: rs110448978 for unclassified [*Mogibacteriaceae*] in our study. This region may harbor a gene that affects both unclassified [*Mogibacteriaceae*] and feed efficiency, or the QTL may contain several linked genes that individually or simultaneously influence these two traits. In addition, it is also possible that host QTLs impact feed efficiency through effects on rumen microbial composition. Further studies are required to confirm these cause-and-effect relationships behind these pleiotropic effects between rumen microbiota and feed efficiency.

Analyzing rumen microbiota estimated using 16S rRNA gene amplicon sequencing, in both dairy cattle [5] and the present study, revealed similar heritable rumen microbial taxa, such as unclassified *Victivallaceae* (*h*^*2*^ = 0.2 in both studies) and unclassified BS11 (*h*^*2*^ = 0.11 reported in [5] vs. *h*^*2*^ = 0.25 in this study), even though these two independent studies were based on different cattle breeds, geographical locations, DNA isolation methods, PCR primers, sequencing process, bioinformatic pipelines, statistic models, and so on. The consistent findings of these two studies not only provided us with stronger biological evidence of host additive genetic effects on rumen microbiota, but also confirmed the technical feasibility to conduct quantitative genetic analysis for gut microbial profiles obtained from a PCR-based approach. It is important to be aware that gut microbial profiles generated from a PCR-based approach may be biased and not truly quantitative due to primer selection [70] and/or amplification condition [71]. Therefore, sequencing PCR amplicons of marker genes is not the ideal strategy to profile the gut microbiota to be used for heritability estimation, GWAS, or other quantitative genetic analysis. To better estimate the host genetic effects on rumen microbiome, PCR-free metagenomics is recommended for future studies as it represents a more accurate strategy for both compositional and functional levels.

In the meantime, it is worth mentioning that analyzing the rumen bacterial community at the species and/or strain level will be more biologically relevant, as microorganisms from the same species/strain may share the same ecological niche and thus perform similar functions in the rumen. However, the existing OTU-based 16S rRNA gene sequencing analysis may not generate convenient and reliable taxonomic classification at bacterial species level, as previously reviewed [72]. Briefly, a certain OTU (> 97% similarity) may contain amplicons from different species, while different OTUs may actually represent amplicons from the same species but multiple gene copies [72]. Due to this technical limitation, both Henderson et al. [15] and the current study analyzed the rumen bacterial community at the genus level, which is one of the limitations in the current study. Potentially, the on-going Hungate 1000 project [51] and the 913 metagenome-assembled genomes [50] will serve as a valuable reference dataset for both marker-gene-based analysis and metagenomic-based approach in future studies, which could enhance the resolution of rumen microbial profiling and help us better understand interactions between host genetics and rumen microorganisms.

## Conclusions

This study assessed the determinant factors for the rumen microbiota, estimated the heritability of rumen microbial taxonomic features, and identified genetic components associated with specific rumen microbial taxa using samples collected from a large cohort of beef cattle (*n* = 709). Rumen microbiota of these beef cattle are generally consistent with those typically described previously at various taxonomic levels [15, 73]. Multiple factors, including breed, sex, and diet were identified to drive the variation of rumen microbiota among animals. The findings on moderate heritability estimates for rumen microbial taxonomic features and the identified microbial taxa associated SNPs from GWAS show direct evidence that rumen microbial colonization in beef cattle can be affected by host additive genetic effects and genotypes. In addition, heritable rumen microbial features were associated with host feed efficiency and rumen VFAs, and there were SNPs associated with both rumen microbiota and feed efficiency. Therefore, cattle may genetically control their rumen microbiota and consequently influence their rumen fermentation and feed efficiency. It is noticeable that when commercial cattle populations were tested, it is challenging to strictly control the diet for every individual, due to breed, sex, and/or environmental (farm) factors. Although both the previous study for dairy cows [5] and our current study for beef cattle identified the host genetic effect on rumen microbiota, future studies with optimized experimental design to provide an identical diet to all the beef cattle are necessary, which may give us more accurate heritability estimates and more convincing associations between bovine genotypes and rumen microbiota. Regardless, together with Difford et al. [5], the findings on host genetics associated rumen microorganisms suggest the potential to manipulate these heritable microbial taxonomic features through genetic selection and breeding, and it could be a useful strategy to optimize rumen fermentation and further improve feed efficiency as well as other rumen microbiota-related traits (e.g., CH_4_ emissions, milk composition, ruminal acidosis, etc.) through targeting both hosts and their rumen microbiota. In addition, to manipulate those environmentally determined phylotypes with low heritability estimates (such as members belonging to *Bacteroidetes* and most of archaeal taxa), individual feeding schemes could be considered. Therefore, it is important to combine both genetics-based (selection and breeding) and management-based (precision feeding schemes) approaches to achieve optimal host-microbiota-diet interactions and thus enhanced the productivity of beef cattle to address the emerging global food security challenges.

## Additional files

Additional file 1: **Table S1.** Phenotype records and metadata for each individual. (XLSX 171 kb)

Additional file 2: **Table S2.** Diet information for animal experiments. (DOCX 17 kb)

Additional file 3: **Table S3.** Feed efficiency traits and VFA concentrations for this beef cattle population. (DOCX 17 kb)

Additional file 4: **Table S4.** Single nucleotide polymorphisms (SNPs) information. (DOCX 18 kb)

Additional file 5: **Table S5.** Alpha-diversity indices of this beef cattle population. (DOCX 17 kb)

Additional file 6: **Table S6.** Relative abundance and heritability estimates of detected rumen microbial taxa, and factors (breed, sex, diet, and age) driving their variation. (XLSX 25 kb)

Additional file 7: **Figure S1.** Microbiota-associated SNPs contribute to the variation of feed efficiency. (DOCX 477 kb)

## Data Availability

All sequencing data are available from the National Center for Biotechnology Information (NCBI) Sequence Read Archive (SRA) under accession number PRJNA393057.

## References

[1] Firkins JL, Yu Z (2015). Ruminant nutrition symposium: how to use data on the rumen microbiome to improve our understanding of ruminant nutrition. J Anim Sci.

[2] Li F, Guan LL. Metatranscriptomic profiling reveals linkages between the active rumen microbiome and feed efficiency in beef cattle. Appl Environ Microbiol. 2017;83. 10.1128/AEM.00061-17. 10.1128/AEM.00061-17 PMC5394315 28235871

[3] Shabat Sheerli Kruger Ben, Sasson Goor, Doron-Faigenboim Adi, Durman Thomer, Yaacoby Shamay, Berg Miller Margret E, White Bryan A, Shterzer Naama, Mizrahi Itzhak (2016). Specific microbiome-dependent mechanisms underlie the energy harvest efficiency of ruminants. The ISME Journal.

[4] Wallace RJ, Rooke JA, McKain N, Duthie CA, Hyslop JJ, Ross DW, Waterhouse A, Watson M, Roehe R (2015). The rumen microbial metagenome associated with high methane production in cattle. BMC Genomics.

[5] Difford GF, Plichta DR, Lovendahl P, Lassen J, Noel SJ, Hojberg O, Wright AG, Zhu Z, Kristensen L, Nielsen HB (2018). Host genetics and the rumen microbiome jointly associate with methane emissions in dairy cows. PLoS Genet.

[6] Jami E, White BA, Mizrahi I (2014). Potential role of the bovine rumen microbiome in modulating milk composition and feed efficiency. PLoS One.

[7] McCann JC, Luan S, Cardoso FC, Derakhshani H, Khafipour E, Loor JJ (2016). Induction of subacute ruminal acidosis affects the ruminal microbiome and epithelium. Front Microbiol.

[8] Spor A, Koren O, Ley R (2011). Unravelling the effects of the environment and host genotype on the gut microbiome. Nat Rev Microbiol.

[9] Benson AK, Kelly SA, Legge R, Ma F, Low SJ, Kim J, Zhang M, Oh PL, Nehrenberg D, Hua K (2010). Individuality in gut microbiota composition is a complex polygenic trait shaped by multiple environmental and host genetic factors. Proc Natl Acad Sci U S A.

[10] Leamy LJ, Kelly SA, Nietfeldt J, Legge RM, Ma F, Hua K, Sinha R, Peterson DA, Walter J, Benson AK, Pomp D (2014). Host genetics and diet, but not immunoglobulin A expression, converge to shape compositional features of the gut microbiome in an advanced intercross population of mice. Genome Biol.

[11] Goodrich JK, Waters JL, Poole AC, Sutter JL, Koren O, Blekhman R, Beaumont M, Van Treuren W, Knight R, Bell JT (2014). Human genetics shape the gut microbiome. Cell.

[12] Goodrich JK, Davenport ER, Beaumont M, Jackson MA, Knight R, Ober C, Spector TD, Bell JT, Clark AG, Ley RE (2016). Genetic determinants of the gut microbiome in UK twins. Cell Host Microbe.

[13] Turpin W, Espin-Garcia O, Xu W, Silverberg MS, Kevans D, Smith MI, Guttman DS, Griffiths A, Panaccione R, Otley A (2016). Association of host genome with intestinal microbial composition in a large healthy cohort. Nat Genet.

[14] Guan LL, Nkrumah JD, Basarab JA, Moore SS (2008). Linkage of microbial ecology to phenotype: correlation of rumen microbial ecology to cattle's feed efficiency. FEMS Microbiol Lett.

[15] Henderson G, Cox F, Ganesh S, Jonker A, Young W, Janssen PH (2015). Rumen microbial community composition varies with diet and host, but a core microbiome is found across a wide geographical range. Sci Rep.

[16] Hernandez-Sanabria E, Goonewardene LA, Wang Z, Zhou M, Moore SS, Guan LL (2013). Influence of sire breed on the interplay among rumen microbial populations inhabiting the rumen liquid of the progeny in beef cattle. PLoS One.

[17] Roehe R, Dewhurst RJ, Duthie CA, Rooke JA, McKain N, Ross DW, Hyslop JJ, Waterhouse A, Freeman TC, Watson M, Wallace RJ (2016). Bovine host genetic variation influences rumen microbial methane production with best selection criterion for Low methane emitting and efficiently feed converting hosts based on metagenomic gene abundance. PLoS Genet.

[18] Paz HA, Anderson CL, Muller MJ, Kononoff PJ, Fernando SC (2016). Rumen bacterial community composition in Holstein and Jersey cows is different under same dietary condition and is not affected by sampling method. Front Microbiol.

[19] Li F, Hitch TCA, Chen Y, Creevey CJ, Guan LL (2019). Comparative metagenomic and metatranscriptomic analyses reveal the breed effect on the rumen microbiome and its associations with feed efficiency in beef cattle. Microbiome.

[20] Jewell KA, McCormick CA, Odt CL, Weimer PJ, Suen G (2015). Ruminal bacterial community composition in dairy cows is dynamic over the course of two lactations and correlates with feed efficiency. Appl Environ Microbiol.

[21] Jami Elie, Israel Adi, Kotser Assaf, Mizrahi Itzhak (2013). Exploring the bovine rumen bacterial community from birth to adulthood. The ISME Journal.

[22] Nkrumah JD, Crews DH, Basarab JA, Price MA, Okine EK, Wang Z, Li C, Moore SS (2007). Genetic and phenotypic relationships of feeding behavior and temperament with performance, feed efficiency, ultrasound, and carcass merit of beef cattle. J Anim Sci.

[23] Olfert ED, Cross BM, McWilliams AA (1993). Guide to the care and use of experimental steers.

[24] Hernandez-Sanabria E, Guan LL, Goonewardene LA, Li M, Mujibi DF, Stothard P, Moore SS, Leon-Quintero MC (2010). Correlation of particular bacterial PCR-denaturing gradient gel electrophoresis patterns with bovine ruminal fermentation parameters and feed efficiency traits. Appl Environ Microbiol.

[25] Basarab JA, Colazo MG, Ambrose DJ, Novak S, McCartney D, Baron VS (2011). Residual feed intake adjusted for backfat thickness and feeding frequency is independent of fertility in beef heifers. Can J Anim Sci.

[26] Stevenson DM, Weimer PJ (2007). Dominance of Prevotella and low abundance of classical ruminal bacterial species in the bovine rumen revealed by relative quantification real-time PCR. Appl Microbiol Biotechnol.

[27] Zhou M, Hernandez-Sanabria E, Le LG (2009). Assessment of the microbial ecology of ruminal methanogens in cattle with different feed efficiencies. Appl Environ Microbiol.

[28] Edgar RC, Haas BJ, Clemente JC, Quince C, Knight R (2011). UCHIME improves sensitivity and speed of chimera detection. Bioinformatics.

[29] Edgar RC (2010). Search and clustering orders of magnitude faster than BLAST. Bioinformatics.

[30] McDonald Daniel, Price Morgan N, Goodrich Julia, Nawrocki Eric P, DeSantis Todd Z, Probst Alexander, Andersen Gary L, Knight Rob, Hugenholtz Philip (2011). An improved Greengenes taxonomy with explicit ranks for ecological and evolutionary analyses of bacteria and archaea. The ISME Journal.

[31] Seedorf H, Kittelmann S, Henderson G, Janssen PH (2014). RIM-DB: a taxonomic framework for community structure analysis of methanogenic archaea from the rumen and other intestinal environments. PeerJ.

[32] Altschul SF, Gish W, Miller W, Myers EW, Lipman DJ (1990). Basic local alignment search tool. J Mol Biol.

[33] Friedman J, Alm EJ (2012). Inferring correlation networks from genomic survey data. PLoS Comput Biol.

[34] Schloss PD, Westcott SL, Ryabin T, Hall JR, Hartmann M, Hollister EB, Lesniewski RA, Oakley BB, Parks DH, Robinson CJ (2009). Introducing mothur: open-source, platform-independent, community-supported software for describing and comparing microbial communities. Appl Environ Microbiol.

[35] Ramayo-Caldas Yuliaxis, Mach Nuria, Lepage Patricia, Levenez Florence, Denis Catherine, Lemonnier Gaetan, Leplat Jean-Jacques, Billon Yvon, Berri Mustapha, Doré Jöel, Rogel-Gaillard Claire, Estellé Jordi (2016). Phylogenetic network analysis applied to pig gut microbiota identifies an ecosystem structure linked with growth traits. The ISME Journal.

[36] Shannon P, Markiel A, Ozier O, Baliga NS, Wang JT, Ramage D, Amin N, Schwikowski B, Ideker T (2003). Cytoscape: a software environment for integrated models of biomolecular interaction networks. Genome Res.

[37] Wimmer V, Albrecht T, Auinger HJ, Schon CC (2012). Synbreed: a framework for the analysis of genomic prediction data using R. Bioinformatics.

[38] VanRaden PM (2008). Efficient methods to compute genomic predictions. J Dairy Sci.

[39] Gilmour AR, Gogel BJ, Cullis BR, Welham SJ, Thompson R, Butler D, Cherry M, Collins D, Dutkowski G, Harding SA (2014). ASReml user guide. Release 4.1 structural specification.

[40] Nicolazzi EL, Caprera A, Nazzicari N, Cozzi P, Strozzi F, Lawley C, Pirani A, Soans C, Brew F, Jorjani H (2015). SNPchiMp v.3: integrating and standardizing single nucleotide polymorphism data for livestock species. BMC Genomics.

[41] Endelman JB (2011). Ridge regression and other kernels for genomic selection with R package rrBLUP. Plant Genome.

[42] Benjamini Y, Hochberg Y (1995). Controlling the false discovery rate: a practical and powerful approach to multiple testing. J R Stat Soc Ser B Methodol.

[43] Core R (2014). Team: R: a language and environment for statistical computing.

[44] Asnicar F, Weingart G, Tickle TL, Huttenhower C, Segata N (2015). Compact graphical representation of phylogenetic data and metadata with GraPhlAn. PeerJ.

[45] Davenport ER, Cusanovich DA, Michelini K, Barreiro LB, Ober C, Gilad Y (2015). Genome-wide association studies of the human gut microbiota. PLoS One.

[46] Org E, Mehrabian M, Parks BW, Shipkova P, Liu X, Drake TA, Lusis AJ (2016). Sex differences and hormonal effects on gut microbiota composition in mice. Gut Microbes.

[47] Yurkovetskiy L, Burrows M, Khan AA, Graham L, Volchkov P, Becker L, Antonopoulos D, Umesaki Y, Chervonsky AV (2013). Gender bias in autoimmunity is influenced by microbiota. Immunity.

[48] Li T, Chiang JY (2015). Bile acids as metabolic regulators. Curr Opin Gastroenterol.

[49] Asnicar F, Manara S, Zolfo M, Truong DT, Scholz M, Armanini F, Ferretti P, Gorfer V, Pedrotti A, Tett A, Segata N. Studying vertical microbiome transmission from mothers to infants by strain-level metagenomic profiling. mSystems. 2017;2. 10.1128/mSystems.00164-16. 10.1128/mSystems.00164-16 PMC5264247 28144631

[50] Stewart RD, Auffret MD, Warr A, Wiser AH, Press MO, Langford KW, Liachko I, Snelling TJ, Dewhurst RJ, Walker AW (2018). Assembly of 913 microbial genomes from metagenomic sequencing of the cow rumen. Nat Commun.

[51] Seshadri R, Leahy SC, Attwood GT, Teh KH, Lambie SC, Cookson AL, Eloe-Fadrosh EA, Pavlopoulos GA, Hadjithomas M, Varghese NJ (2018). Cultivation and sequencing of rumen microbiome members from the Hungate1000 collection. Nat Biotechnol.

[52] David LA, Maurice CF, Carmody RN, Gootenberg DB, Button JE, Wolfe BE, Ling AV, Devlin AS, Varma Y, Fischbach MA (2014). Diet rapidly and reproducibly alters the human gut microbiome. Nature.

[53] Sasson G, Kruger Ben-Shabat S, Seroussi E, Doron-Faigenboim A, Shterzer N, Yaacoby S, Berg Miller ME, White BA, Halperin E, Mizrahi I. Heritable bovine rumen Bacteria are phylogenetically related and correlated with the cow’s capacity to harvest energy from its feed. MBio. 2017;8. 10.1128/mBio.00703-17. 10.1128/mBio.00703-17 PMC5559629 28811339

[54] Russell JB, Rychlik JL (2001). Factors that alter rumen microbial ecology. Science.

[55] Klieve AV, O'Leary MN, McMillen L, Ouwerkerk D (2007). Ruminococcus bromii, identification and isolation as a dominant community member in the rumen of cattle fed a barley diet. J Appl Microbiol.

[56] Huws SA, Kim EJ, Lee MR, Scott MB, Tweed JK, Pinloche E, Wallace RJ, Scollan ND (2011). As yet uncultured bacteria phylogenetically classified as Prevotella, Lachnospiraceae incertae sedis and unclassified Bacteroidales, Clostridiales and Ruminococcaceae may play a predominant role in ruminal biohydrogenation. Environ Microbiol.

[57] La Reau AJ, Meier-Kolthoff JP, Suen G (2016). Sequence-based analysis of the genus Ruminococcus resolves its phylogeny and reveals strong host association. Microb Genom.

[58] Pope PB, Smith W, Denman SE, Tringe SG, Barry K, Hugenholtz P, McSweeney CS, McHardy AC, Morrison M (2011). Isolation of Succinivibrionaceae implicated in low methane emissions from Tammar wallabies. Science.

[59] Li F, Henderson G, Sun X, Cox F, Janssen PH, Guan le L (2016). Taxonomic assessment of rumen microbiota using total RNA and targeted amplicon sequencing approaches. Front Microbiol.

[60] Kong RS, Liang G, Chen Y, Stothard P, Guan le L (2016). Transcriptome profiling of the rumen epithelium of beef cattle differing in residual feed intake. BMC Genomics.

[61] Aschenbach JR, Penner GB, Stumpff F, Gabel G (2011). Ruminant nutrition symposium: role of fermentation acid absorption in the regulation of ruminal pH. J Anim Sci.

[62] Hernandez J, Benedito JL, Abuelo A, Castillo C (2014). Ruminal acidosis in feedlot: from aetiology to prevention. ScientificWorldJournal.

[63] Xiang R, McNally J, Rowe S, Jonker A, Pinares-Patino CS, Oddy VH, Vercoe PE, McEwan JC, Dalrymple BP (2016). Gene network analysis identifies rumen epithelial cell proliferation, differentiation and metabolic pathways perturbed by diet and correlated with methane production. Sci Rep.

[64] Racca AW, Beck AE, McMillin MJ, Korte FS, Bamshad MJ, Regnier M (2015). The embryonic myosin R672C mutation that underlies Freeman-Sheldon syndrome impairs cross-bridge detachment and cycling in adult skeletal muscle. Hum Mol Genet.

[65] de Oliveira PS, Cesar AS, do Nascimento ML, Chaves AS, Tizioto PC, Tullio RR, Lanna DP, Rosa AN, Sonstegard TS, Mourao GB (2014). Identification of genomic regions associated with feed efficiency in Nelore cattle. BMC Genet.

[66] Li C, Basarab J, Snelling WM, Benkel B, Murdoch B, Moore SS (2002). The identification of common haplotypes on bovine chromosome 5 within commercial lines of Bos taurus and their associations with growth traits. J Anim Sci.

[67] Sherman EL, Nkrumah JD, Li C, Bartusiak R, Murdoch B, Moore SS (2009). Fine mapping quantitative trait loci for feed intake and feed efficiency in beef cattle. J Anim Sci.

[68] Rolf MM, Taylor JF, Schnabel RD, McKay SD, McClure MC, Northcutt SL, Kerley MS, Weaber RL (2012). Genome-wide association analysis for feed efficiency in Angus cattle. Anim Genet.

[69] Myer PR, Smith TP, Wells JE, Kuehn LA, Freetly HC (2015). Rumen microbiome from steers differing in feed efficiency. PLoS One.

[70] Hong SunHee, Bunge John, Leslin Chesley, Jeon Sunok, Epstein Slava S (2009). Polymerase chain reaction primers miss half of rRNA microbial diversity. The ISME Journal.

[71] Huber JA, Morrison HG, Huse SM, Neal PR, Sogin ML, Mark Welch DB (2009). Effect of PCR amplicon size on assessments of clone library microbial diversity and community structure. Environ Microbiol.

[72] Li F, Neves ALA, Ghoshal B, Guan LL (2018). Symposium review: mining metagenomic and metatranscriptomic data for clues about microbial metabolic functions in ruminants. J Dairy Sci.

[73] Kim M, Yu Z (2014). Variations in 16S rRNA-based microbiome profiling between pyrosequencing runs and between pyrosequencing facilities. J Microbiol.

