# Supplementary material for: Host genetics influence the rumen microbiota and heritable rumen microbial features associate with feed efficiency in cattle
Source: Microbiome. 2019 Jun 13;7:92. doi: 10.1186/s40168-019-0699-1 (PMC6567441; doi:10.1186/s40168-019-0699-1)
Supplement: Supplementary file 2 — Table S2. Diet information for animal experiments. (DOCX 17 kb) [file 40168_2019_699_MOESM2_ESM.docx]

**Table S2.** Diet information for animal experiments

| Sex | Breed^1^ | Diet | Number of animals | Diet composition |
| --- | --- | --- | --- | --- |
| Steer | HYB | Diet 1 | 161 | 95% straight oats and free choice hay, 5% rumensin pellet^2^ |
|  | CHAR | Diet 1 | 50 |  |
|  | ANG | Diet 2 | 80 | Straight hay |
|  |  |  |  |  |
| Bull | HYB | Diet 3 | 42 | 80% Silage, 15% whole oats, 5% rumensin pellet^2^ |
|  | CHAR | Diet 3 | 9 |  |
|  | ANG | Diet 3 | 20 |  |
|  |  |  |  |  |
| Heifer | HYB | Diet 4 | 189 | 55% Silage, 40% whole oats, 5% rumensin pellet^2^ |
|  | ANG | Diet 4 | 103 |  |
|  | CHAR | Diet 1 | 55 | 95% straight oats and free choice hay, 5% rumensin pellet^2^ |

***Note:*** ^1^ HYB = Kinsella composite hybrid, CHAR = Charolais, and ANG = Angus.

^2^ Rumensin pellet = Killam 30% Beef Supplement Pellets (Tag 849053; Hi-Pro Feeds, Westlock, AB, Canada).
