# Supplementary material for: Host genetics influence the rumen microbiota and heritable rumen microbial features associate with feed efficiency in cattle
Source: Microbiome. 2019 Jun 13;7:92. doi: 10.1186/s40168-019-0699-1 (PMC6567441; doi:10.1186/s40168-019-0699-1)
Supplement: Supplementary file 3 — Table S3. Feed efficiency traits and VFA concentrations for this beef cattle population. (DOCX 17 kb) [file 40168_2019_699_MOESM3_ESM.docx]

**Table S3.** Feed efficiency traits and VFA concentrations for this beef cattle population

| **Trait** |  | Number of animals | mean ± SEM |
| --- | --- | --- | --- |
| DMI (dry matter intake; kg/day) |  | 572 | 9.23 ± 0.06 |
| ADG (average daily gain; kg/day) |  | 572 | 1.28 ± 0.02 |
| FCR (feed conversion ratio) |  | 572 | 7.60 ± 0.07 |
| RFI (Residual feed intake; kg/day) |  | 572 | 0.00 ± 0.02 |
| RFIf (backfat-adjusted RFI; kg/day) |  | 572 | 0.00 ± 0.02 |
| Total volatile fatty acids (mM) |  | 708 | 60.48 ± 0.60 |
| Acetate (%) |  | 708 | 62.05 ± 0.25 |
| Propionate (%) |  | 708 | 23.44 ± 0.25 |
| Butyrate (%) |  | 708 | 9.93 ± 0.11 |
| Isobutyrate (%) |  | 708 | 1.09 ± 0.01 |
| Valerate (%) |  | 708 | 1.13 ± 0.02 |
| Isovalerate (%) |  | 708 | 2.05 ± 0.04 |
| Caproate (%) |  | 708 | 0.32 ± 0.01 |
