# Supplementary material for: Host genetics influence the rumen microbiota and heritable rumen microbial features associate with feed efficiency in cattle
Source: Microbiome. 2019 Jun 13;7:92. doi: 10.1186/s40168-019-0699-1 (PMC6567441; doi:10.1186/s40168-019-0699-1)
Supplement: Supplementary file 4 — Table S4. Single nucleotide polymorphisms (SNPs) information. (DOCX 18 kb) [file 40168_2019_699_MOESM4_ESM.docx]

**Table S4.** Single nucleotide polymorphisms (SNPs) information

| **BTA** | **SNP^1^**  **(n)** | **SNP interval**  **(Mean, bp)** |  | **BTA** | **SNP^1^** | **SNP interval**  **(Mean, bp)** |
| --- | --- | --- | --- | --- | --- | --- |
| 1 | 2739 | 57741 |  | 16 | 1348 | 60253 |
| 2 | 2265 | 60363 |  | 17 | 1333 | 56221 |
| 3 | 2049 | 59151 |  | 18 | 1087 | 60222 |
| 4 | 2045 | 58885 |  | 19 | 1145 | 55519 |
| 5 | 1744 | 69466 |  | 20 | 1296 | 55146 |
| 6 | 2104 | 56607 |  | 21 | 1141 | 62367 |
| 7 | 1850 | 60766 |  | 22 | 1050 | 58357 |
| 8 | 1958 | 57695 |  | 23 | 889 | 58812 |
| 9 | 1700 | 62074 |  | 24 | 1058 | 58753 |
| 10 | 1788 | 58295 |  | 25 | 803 | 53258 |
| 11 | 1810 | 59224 |  | 26 | 899 | 56741 |
| 12 | 1391 | 65428 |  | 27 | 781 | 58015 |
| 13 | 1461 | 57440 |  | 28 | 812 | 56946 |
| 14 | 1472 | 56528 |  | 29 | 882 | 58005 |
| 15 | 1378 | 61325 |  | X | 96 | 1150102 |
| #SNPs before quality control filter: 54609  #SNPs not following Hardy-Weinberg equilibrium: 1802  #SNPs with minor allele frequency (MAF) < 5% and genotyping call rate < 90%: 9998  #Available SNPs for genomic relationship matrix construction: 42809  #SNPs without position information: 435  #Available SNPs for genome wide association study: 42374 | | | | | | |

***Note****:* ^1^ These numbers are the SNPs involved into the genome wide association study.
