# Supplementary material for: Host genetics influence the rumen microbiota and heritable rumen microbial features associate with feed efficiency in cattle
Source: Microbiome. 2019 Jun 13;7:92. doi: 10.1186/s40168-019-0699-1 (PMC6567441; doi:10.1186/s40168-019-0699-1)
Supplement: Supplementary file 5 — Table S5. Alpha-diversity indices of this beef cattle population. (DOCX 17 kb) [file 40168_2019_699_MOESM5_ESM.docx]

**Table S5.** Alpha-diversity indices^1^ of this beef cattle population

| Al | **Bacterial community^2^** | | |  | **Archaeal community^2^** | | |
| --- | --- | --- | --- | --- | --- | --- | --- |
|  | Mean | SEM | CV (%) |  | Mean | SEM | CV (%) |
|  |  | (n = 658) |  |  |  | (n = 660) |  |
| Chao1 | 60.88 | 0.48 | 20.06 |  | 6.48 | 0.09 | 37.26 |
| Shannon | 3.00 | 0.02 | 15.60 |  | 0.63 | 0.02 | 62.72 |
| Simpson | 0.78 | 0.00 | 12.63 |  | 0.21 | 0.01 | 73.40 |
| Good's coverage | 99.33% | 0.00 | 0.15 |  | 99.72% | 0.00 | 0.14 |

***Note:*** ^1^ To estimate these α-diversity indices, the number of bacterial and archaeal sequences per sample were normalized to 2,000 and 500, respectively, using 100 subsampling iterations.

^2^ These α-diversity indices were calculated at the genus level for bacterial communities, and at the species level for archaeal communities.
