# Supplementary material for: Host genetics influence the rumen microbiota and heritable rumen microbial features associate with feed efficiency in cattle
Source: Microbiome. 2019 Jun 13;7:92. doi: 10.1186/s40168-019-0699-1 (PMC6567441; doi:10.1186/s40168-019-0699-1)
Supplement: Supplementary file 7 — Figure S1. Microbiota-associated SNPs contribute to the variation of feed efficiency. (DOCX 477 kb) [file 40168_2019_699_MOESM7_ESM.docx]

**Fig. S1** Microbiota-associated SNPs contribute to the variation of feed efficiency. Each dot represents feed efficiency for a genotype and expressed as mean ± SE. *P* values among different SNP genotypes were obtained using ANOVA, and values that do not have a common superscript are different with *P* < 0.10.
